# A network approach to prioritize conservation efforts for migratory birds

**DOI:** 10.1111/cobi.13383

**Published:** 2019-08-16

**Authors:** Yanjie Xu, Yali Si, John Takekawa, Qiang Liu, Herbert H. T. Prins, Shenglai Yin, Diann J. Prosser, Peng Gong, Willem F. de Boer

**Affiliations:** ^1^ Ministry of Education Key Laboratory for Earth System Modelling and Department of Earth System Science Tsinghua University 30 Shuangqing Road Beijing 100084 China; ^2^ Resource Ecology Group Wageningen University and Research Droevendaalsesteeg 3a 6708 PB Wageningen the Netherlands; ^3^ Suisun Resource Conservation District 2544 Grizzly Island Road Suisun City CA 94585 U.S.A.; ^4^ Network Architectures and Services Group Delft University of Technology Mekelweg 4 2600 GA Delft the Netherlands; ^5^ U.S. Geological Survey Patuxent Wildlife Research Centre Laurel MD 20708 U.S.A.

**Keywords:** bird migration, connectivity, conservation designation, habitat loss, network, conectividad, designación de conservación, migración de aves, pérdida de hábitat, redes, 鸟类迁徙, 网络, 保护设定, 连通性, 栖息地丧失

## Abstract

Habitat loss can trigger migration network collapse by isolating migratory bird breeding grounds from nonbreeding grounds. Theoretically, habitat loss can have vastly different impacts depending on the site's importance within the migratory corridor. However, migration‐network connectivity and the impacts of site loss are not completely understood. We used GPS tracking data on 4 bird species in the Asian flyways to construct migration networks and proposed a framework for assessing network connectivity for migratory species. We used a node‐removal process to identify stopover sites with the highest impact on connectivity. In general, migration networks with fewer stopover sites were more vulnerable to habitat loss. Node removal in order from the highest to lowest degree of habitat loss yielded an increase of network resistance similar to random removal. In contrast, resistance increased more rapidly when removing nodes in order from the highest to lowest betweenness value (quantified by the number of shortest paths passing through the specific node). We quantified the risk of migration network collapse and identified crucial sites by first selecting sites with large contributions to network connectivity and then identifying which of those sites were likely to be removed from the network (i.e., sites with habitat loss). Among these crucial sites, 42% were not designated as protected areas. Setting priorities for site protection should account for a site's position in the migration network, rather than only site‐specific characteristics. Our framework for assessing migration‐network connectivity enables site prioritization for conservation of migratory species.

## Abstract 2

*Article impact statement*: Analysis of migration‐network connectivity enables the prioritization for site conservation.

## Abstract (es)

Un Enfoque de Redes para Priorizar los Esfuerzos de Conservación para las Aves Migratorias

**Resumen**

La pérdida del hábitat puede disparar el colapso de las redes de migración al aislar los sitios de reproducción de las aves migratorias de aquellos sitios que no se usan para la reproducción. En teoría, la pérdida del hábitat puede tener impactos muy diferentes dependiendo de la importancia del sitio dentro del corredor migratorio. Sin embargo, la conectividad entre las redes de migración y los impactos de la pérdida de los sitios no están del todo comprendidos. Usamos los datos de seguimiento por GPS de cuatro especies de aves en las rutas de vuelo de Asia para construir redes de migración y propusimos un marco de trabajo para evaluar la conectividad de las redes en las especies migratorias. Usamos un proceso de extracción de nodos para identificar los sitios de escala con el mayor impacto sobre la conectividad. En general, las redes de migración con menos sitios de escala fueron más vulnerables a la pérdida del hábitat. La extracción de nodos en orden del grado más alto al más bajo resultó en un incremento de resistencia de la red similar a la extracción al azar. Al contrario, la resistencia incrementó más rápidamente cuando la extracción de los nodos fue en orden del más alto al más bajo valor de intermediación (cuantificado por el número de caminos más cortos que pasan por un nodo específico). Cuantificamos el riesgo de colapso de la red de migración e identificamos sitios cruciales al seleccionar primero los sitios con mayores contribuciones a la conectividad de la red y después identificar cuáles de esos sitios tenían probabilidad de ser removidos de la red (es decir, sitios con pérdida de hábitat). Entre estos sitios cruciales, el 42% no estaban designados como áreas protegidas. El establecimiento de prioridades para la protección de un sitio debería considerar la posición del sitio dentro de la red de migración, en lugar de sólo considerar las características específicas del sitio. Nuestro marco de trabajo para la evaluación de la conectividad de la red de migración permite la priorización de sitios para la conservación de las especies migratorias.

## 摘要

栖息地丧失导致的迁徙鸟类繁殖地和非繁殖地隔离会引发迁徙网络的瓦解。理论上, 取决于该栖息地在迁徙廊道里的重要性, 不同栖息地节点的丧失会对迁徙网络造成不同的影响, 但目前对迁徙网络连通性以及栖息地丧失对迁徙网络影响的理解还不完善。本研究基于亚洲迁徙路线上4 种迁徙鸟类物种的 GPS 追踪数据, 分别构建了迁徙网络并提出了衡量迁徙物种迁徙网络连通性的方法体系。我们使用节点去除法来确定高重要性中途停歇地。具有较少停歇地的迁徙网络通常受栖息地丧失的威胁更严重。我们发现, 依据栖息地丧失程度由高到低的顺序去除栖息地, 迁徙网络阻力的增加趋势与随机去除栖息地结果基本一致。而如果依据栖息地节点的中间值 (表征通过该节点的最短路径数量) 由高到低的顺序去除栖息地节点, 迁徙阻力则呈现快速上升。我们量化了迁徙网络瓦解的风险并基于栖息地节点对迁徙网络连通性的贡献和节点的脆弱性 (栖息地退化程度) 识别了关键栖息地。其中 42% 的关键栖息地处于非保护地状态。我们建议设定栖息地优先保护级别时需要考虑该栖息地在迁徙网络中的位置和作用, 而不是仅依据该栖息地的本身特征。本研究提出的栖息地网络连通性评价体系为迁徙物种栖息地优先保护提供了重要理论和方法依据。

## Introduction

In recent years, the populations of many migratory species have declined rapidly because of loss and degradation of habitats, caused by rapid economic development, intensive human disturbance, and inefficient conservation policies (Syroechkovskiy 2006; de Boer et al. 2011; Studds et al. 2017). The majority of migratory birds are not effectively protected across their migration network (Runge et al. 2014; Dhanjal‐Adams et al. 2017). For instance, the swan goose (*Anser cygnoides*) is categorized as vulnerable, but is substantially threatened by high levels of hunting and wetland conversion (Birdlife International 2016). The greater white‐fronted goose (*Anser albifrons*), bar‐headed goose (*Anser indicus*), and whooper swan (*Cygnus cygnus*) are widespread and abundant in the wild and are categorized as least concern species, but their populations have rapidly declined in many areas, indicating inadequate conservation efforts for these migratory birds (Syroechkovskiy 2006; Si et al. 2018).

Wetland degradation and loss can weaken the integrity of the migration networks of individual species, or even promote migration network collapse, by isolating species’ breeding grounds from wintering grounds (Shimazaki et al. 2004). Moreover, degradation and loss of stepping‐stone nodes from a habitat network may limit a species’ ability to shift ranges, which is an important strategy used by migratory birds to cope with environmental changes (Saura et al. 2014). To better understand how environmental changes affect existing migration networks and to guide targeted conservation measures, it is important to evaluate a migration network's connectivity and resilience and to identify crucial sites that might trigger network collapse.

In conservation policy making, a site's importance for migratory species is often evaluated in terms of the presence of suitable habitat, habitat vulnerability, degree of habitat loss, protection status, and species abundance or diversity (Mehlman et al. 2005; Bayly et al. 2012; Merken et al. 2015). Although such evaluation is straightforward, it does not account for relationships among different sites or the site's context within a network (e.g., the availability of alternative sites along the migration flyway) (Merken et al. 2015). Previous studies demonstrate that network‐level metrics (e.g., habitat centrality) are more suitable for evaluating habitat importance for species with a movement pattern and should thus be included in management decisions (Nicol et al. 2016; Dhanjal‐Adams et al. 2017; Bieri et al. 2018). Recent resolutions emphasize the importance of considering ecological networks and the connectivity of migratory species when addressing conservation of migratory species (UNEP 2017).

Theoretically, the same degree of habitat loss from sites at different network locations could have completely different impacts on migratory birds, ranging from not affecting the population size at all to causing rapid extirpation of the species (Weber et al. 1999; Runge et al. 2014). For instance, rapid population decline has occurred among migratory birds with higher reliance on stopover sites in the Yellow Sea region (Studds et al. 2017), which may be abundant in resources providing energy reserves for subsequent migration (Baker et al. 2004) or located in a critical position connecting breeding and nonbreeding sites (Shimazaki et al. 2004). Thus, in this case, population decline may not be stopped by implementing conservation measures elsewhere (Runge et al. 2014).

Because bird migration is a directed event that occurs at continental or cross‐continent scales, setting priorities for conservation efforts requires an integrated evaluation that considers both habitat availability and connectivity of sites along migration routes. Network theory is useful for such evaluations. Most studies investigating complete bird migration networks are based on theoretical investigations of conceptual site configurations (Weber et al. 1999; Jensen et al. 2008; Iwamura et al. 2013; Hostetler et al. 2015; Sample et al. 2018). To prioritize conservation efforts for specific sites, it is critical to combine node removal scenarios and habitat loss patterns in migration networks that are empirically defined by sites exhibiting seasonal bird occupation.

Merken et al. (2015) analyzed the migration networks along the Black Sea–Mediterranean Flyway across the Sahara, revealing that the trans‐Sahara migration flyway for waterbirds was well connected. Crucial sites in this migration network were identified by quantifying the importance of each involved wetland. Shimazaki et al. (2004) analyzed migration networks of the oriental white stork (*Ciconia boyciana*) and determined potential collapse risks by simulating the removal of important stopover sites. They demonstrated that the storks will be unable to reach their wintering sites along the Yangtze River if they lose stopovers in the Bohai Bay during southward migration. Iwamura et al. (2014) and Nicol et al. (2015) simulated population flows in shorebird migration networks subject to sea‐level rise and thereby provided insightful algorithms of population flows within these migration networks for use in developing efficient conservation strategies. Additional information is needed regarding the extent to which site‐specific variables (e.g., habitat loss) and network metrics that characterize a node with regard to its network position contribute to migration network breakdown.

We investigated how migration‐network connectivity was affected by site‐specific habitat loss and node‐specific network metrics. Using high‐resolution GPS tracking data, we quantified migration‐network connectivity for large‐bodied waterfowl species in the Central and East Asian–Australasian Flyways. We quantified the importance of each stopover site based on its contribution to the network's resistance relative to bird migration. These data, together with the degree of habitat loss and the protection status of these sites, were used to identify sites for which conservation efforts should be prioritized. We sought to inform priority setting for site conservation.

## Methods

### Data

**Figure 1:**
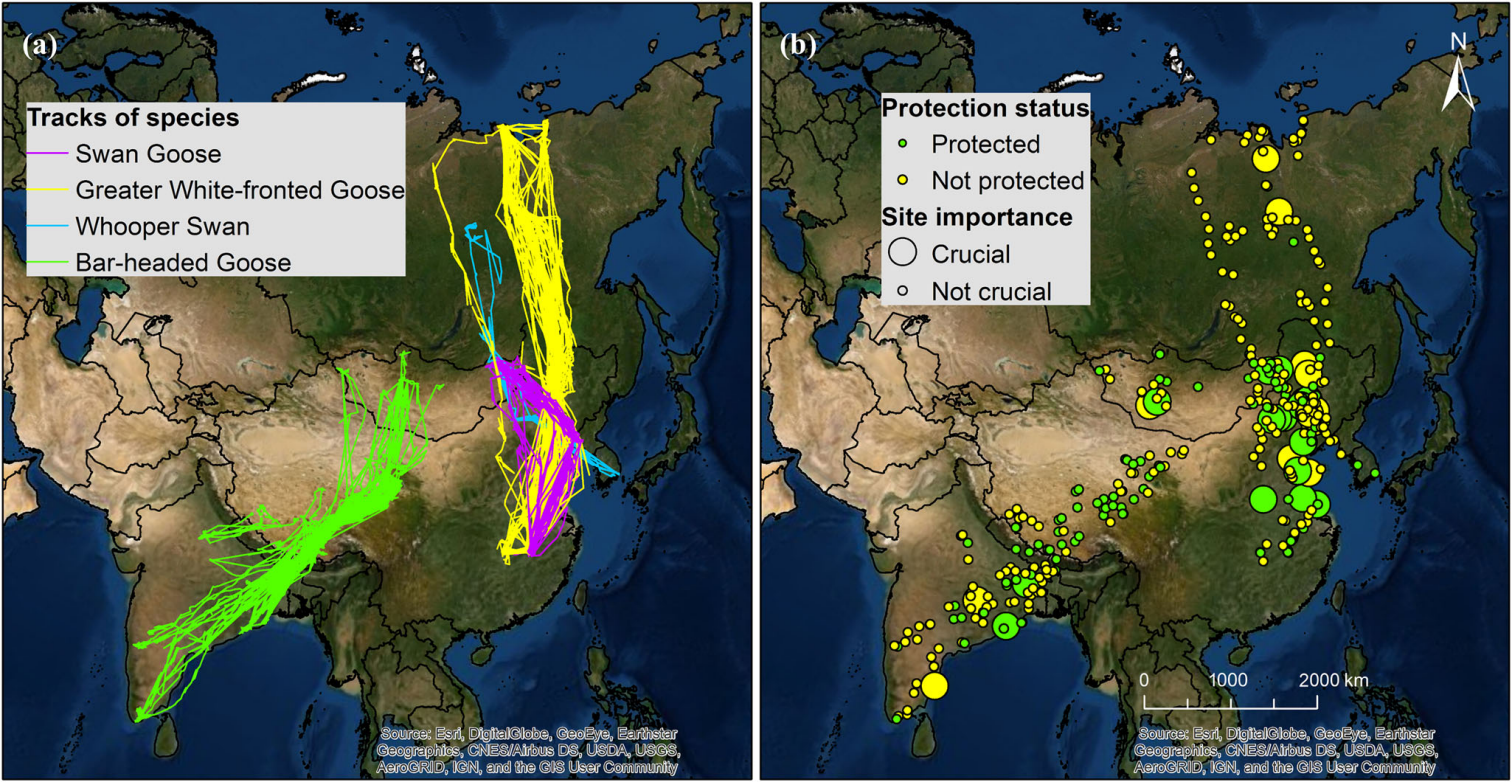
(a) Satellite tracks and (b) breeding, nonbreeding, and stopover sites of swan geese, greater white‐fronted geese, whooper swans, and bar‐headed geese (not protected, site contained no designated protected area; crucial, sites with a normalized betweenness of ≥10% and habitat loss [mean = 7.8%, 95% CI 4.1%]).

A total of 81 swan geese, 54 greater white‐fronted geese, 93 bar‐headed geese, and 10 whooper swans were tagged with GPS loggers in the East Asian–Australasian Flyway and Central Asian Flyway from 2005 to 2018. We obtained 63 full tracks of their northward migration and 108 full tracks of southward migration (Fig. 1a & Supporting Information). The loggers were programed to record 6–12 GPS locations (latitude and longitude) per day for each individual. However, GPS records were sometimes missing due to low battery levels or satellite acquisition failure. Detailed capture and deployment methods are provided in Supporting Information and in Newman et al. (2012), Batbayar et al. (2013), Si et al. (2018), and Xu and Si (2019).

To quantify the degree of habitat loss for each site in migration networks of the focal species, we obtained land‐cover data for 1992 and 2015 from the European Space Agency CCI 300‐m annual global land‐cover products (http://esa-landcover-cci.org). We quantified wetland loss from 1992 to 2015 by extracting the area of water and grassland from the maps of these years. Habitat loss calculation did not include changes in croplands. Although American and European goose populations benefit from agriculture expansion, massive wetland conversion to agricultural lands (Niu et al. 2012) has a negative impact on most waterfowl species in eastern Asia (Si et al. 2018; Xu et al. 2019b).

### Utilized Sites and Migration Lags

We identified the breeding, wintering, and stopover sites of each individual bird with a dynamic Brownian bridge movement model (dBBMM) (Kranstauber et al. 2012). Utilization distributions were derived at a 10 × 10 km resolution for the annual northward and southward migration of each tracked bird. Based on visual inspection of the tracking data, we used a window size of 11 locations and margin size of 3 locations (Kranstauber et al. 2012). Geographical ranges of 90% isopleths of the utilization distributions (i.e., highly utilized areas with short flights) (Si et al. 2018) were defined as the sites utilized in northward and southward migrations (i.e., breeding, nonbreeding, and stopover sites) based on visual inspection. We included sites that birds used for ≥2 days (Si et al. 2018), considering that a site should be used for at least 48 h for settling and refueling (Drent et al. 2006). To measure the effects of sample size, we conducted a sensitivity analysis of the effect of the number of southward tracks of swan geese and bar‐headed geese on site detection (Supporting Information).

We defined migration lag as the nonstop flight distance from one site to the next. Distances between the boundaries of utilized sites were calculated under Azimuthal equidistant projection. Tracks missing data for over 2 weeks were excluded from distance calculations. We calculated the maximum and median migration lags from the tracking data per season, per species.

### Migration Networks

The breeding, nonbreeding, and stopover sites for each individual bird were defined as nodes in the migration network. When the distance between 2 nodes was shorter than the maximum migration lag, these nodes were connected. Due to seasonal directionality, only low‐latitude to high‐latitude sites were connected in northward migration networks and only high‐latitude to low‐latitude sites were connected in southward migration networks. We assumed that greater site‐to‐site distance was associated with increased cost of movements between sites. Thus, the weight of each site‐to‐site connection was defined by a between‐sites dispersal probability, calculated as the cost of moving between 2 sites with a decreasing exponential function (Eq. 1) (Keitt et al. 1997). This probability function assumed that a greater distance between 2 sites correlated with a lower probability that migratory birds would move from one site to the next. For simplicity, this assumption was based only on energetic expenditures without considering differences in searching and settling costs, forage abundance and quality, or predation risk (Dokter et al. 2018). Based on these nodes and weighted connections, we constructed northward and southward migration networks for each species.

(1) $$P_{\mathrm{ij}}=e^{-kd_{ij}}\;,$$

*P_ij_* is the dispersal probability between sites *i* and *j*, *d_ij_* is the edge‐to‐edge distance between sites *i* and *j*; and *k* is a constant defined by the migration lags of the tracked species. We set *k* to obtain a dispersal probability of 50% when *d_ij_* equaled the median migration lag of the focal species.

### Network Metrics

To identify important stepping‐stone sites, we calculated network metrics related to node centrality: betweenness, weighted degree, and node resistance. For each pair of sites in the migration network, we identified the shortest path (i.e., minimum weighted path length between the 2 nodes) via the Dijkstra algorithm (Dijkstra 1959). Node betweenness was quantified by the number of shortest paths passing through that node (Freeman 1979; Brandes 2001) and was calculated using the second‐generation weighted betweenness measure (Opsahl et al. 2010). Node betweenness was normalized by dividing it by the highest betweenness value. Node degree indicates the connection strength between the focal site and other sites in the network and was measured as the sum of weights of the connections to and from the focal node, again calculated with the Dijkstra algorithm (Dijkstra 1959). Node resistance was the effective resistance (McRae et al. 2008) between the focal node (i.e., a stopover site) and the breeding site and between the focal node and the nonbreeding site. Node resistance indicates the resistance for traveling between the focal stopover site to the breeding site and the nonbreeding site. We also calculated the degree of habitat loss at each stopover site as the ratio of habitat loss to gain from 1992 to 2015. We selected 1992 as a baseline due to the rapid urbanization and socioeconomic development in East Asian countries since 1992 (Seto & Fragkias 2005; Xu et al. 2019b). The 1992 data are the earliest land‐cover map in the analyzed data set.

We quantified each metric's importance by comparing its contribution to network connectivity (quantified by effective resistance) in a site‐removal process in which removal order was determined by betweenness, node degree, node resistance, or degree of habitat loss. Because our focus was to identify important stepping‐stone sites connecting breeding and nonbreeding sites, the breeding and nonbreeding sites were not included in the site‐removal process. We calculated the metrics for all sites of the initial networks and then removed 1 site at a time under 5 scenarios: highest to lowest relative betweenness, highest to lowest weighted degree, lowest to highest node resistance, highest to lowest degree of habitat loss, and 99 sequences of random removal (each observed network comprised <99 sites). Effective resistance reflected the connectivity between breeding and nonbreeding sites by accounting for both migration cost and alternative routes (McRae et al. 2008). After each removal, we calculated the network's effective resistance and compared the speed of effective resistance increase under different site removal scenarios.

To select the best metric for defining crucial network sites, we compared the effect index of node removal (*E_m_*) (Eq. 2) with different network metrics (*m =* betweenness, degree, or node resistance).

(2) $$\;E_{m}=\frac{N_{0}\times \ln\left(\frac{R_{\mathrm{mn}}}{RR_{n}}\right)}{N_{c}},$$

where RR*_n_* is the effective resistance when *n* nodes are removed at random, *R_mn_* is the effective resistance when *n* nodes are removed in the sequence of metric *m*, *N*_0_ is the total number of nodes in the original network, and *N_c_* is the number of nodes removed upon network collapse. Because *E_m_* was not normally distributed (Kolmogorov–Smirnov test, *D* = 0.24, *p* < 0.001), we used a Kruskal–Wallis test followed by a nonparametric multiple comparison test to analyze differences in the effects of different metrics (*E*_betweenness_, *E*_degree_ *, E*_node resistance_). The metric with a significantly higher *E_m_* level (*p* ≤ 0.05) was used to define a crucial stopover site. Therefore, we defined a site's importance for maintaining network connectivity based on betweenness to illustrate how sites could be ranked based on our node‐removal approach. We also tested between‐metrics differences for each network separately to reveal species differences (Supporting Information).

To define whether a site was protected, we overlapped the map of protected areas with the ranges of sites in the migration networks. Some Chinese protected areas were missing from the international data set; thus, we merged the polygon map from the World Database of Protected Areas (WDPA) (accessed on 6 April 2018 at protectedplanet.net) with the national protected areas in China (MEP 2009) and the site network of the East Asian–Australasian Flyway Partnership (EAAFP) (accessed 20 May 2019 at eaaflyway.net). When a site overlapped with the map of protected areas, we defined it as protected. Otherwise, it was considered not protected. These calculations were performed in ArcMap 10.2.1 under the cylindrical equal‐area projection.

## Results

### Migration Patterns

Swan geese, greater white‐fronted geese, whooper swans, and bar‐headed geese exhibited northward migration networks comprising 23, 72, 15, and 81 sites and southward migration networks comprising 45, 27, 13, and 67 sites, respectively. The small sample size of whooper swans yielded an artificially small number of migration network nodes (Supporting Information); therefore, whooper swan results were included only as an illustrative example of small networks. Among all tracked birds, the median distance between sites was 203 km in both northward and southward migration. The maximum migration lag (travel distance between sites) was 3180 km for southward migration and 3018 km for northward migration (Supporting Information).

### Site Removal

**Figure 2:**
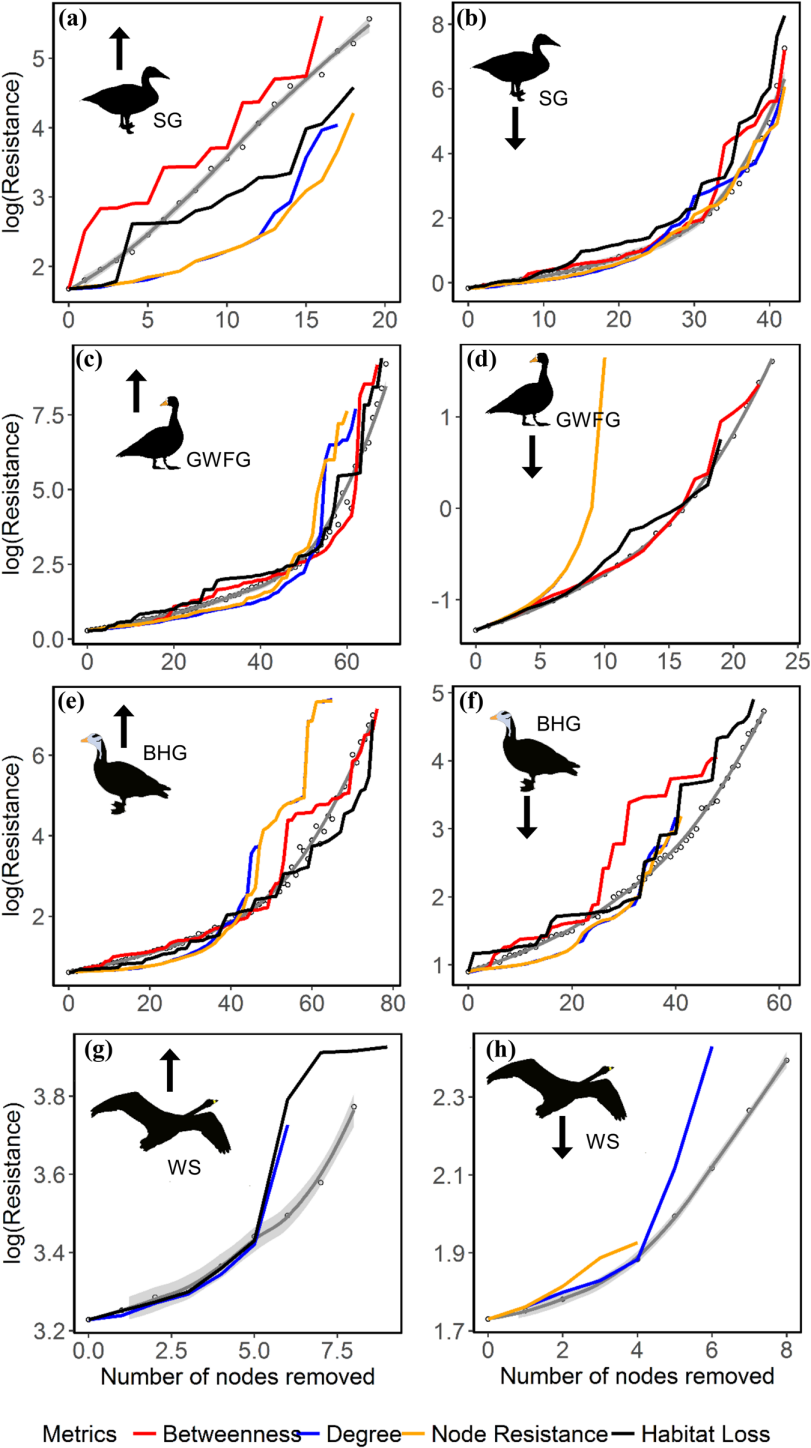
Changes in effective resistance of migration networks on cumulative removal of stopover sites (nodes) of swan geese (SG), greater white‐fronted geese (GWFG), bar‐headed geese (BHG), and whooper swans (WS) (gray lines and dots, migration networks’ effective resistance with random site removal; other lines, changes of effective resistance with site removal in the order of its degree of habitat loss [black], betweenness [red], degree [blue], and node resistance [yellow]; [a], [c], [e], [g], northward migration networks; [b], [d], [f], [h], southward migration networks). Upon network collapse, the effective resistance becomes infinity, such that the end of each line represents the point at which the network collapses. Due to the small number of tracked whooper swans, their data are used only as an example of how a small network behaves.

In general, the migration networks’ effective resistance slowly increased at the beginning of node removal, rising with increasing removal of nodes (Figs. 2b, 2c, 2e, & 2f). However, the effective resistance increase was rapid at the start of node removal in migration networks comprising relatively low numbers of sites for greater white‐fronted geese (southward), swan geese (northward), and whooper swans (both directions) (Figs. 2a, 2d, 2g, & 2h).

Compared with random site removal, the migration networks’ effective resistance generally increased faster when sites were removed in order of increasing betweenness. Small networks (whooper swans) collapsed quickly upon removal of the site with highest betweenness (Figs. 2g & 2h). However, in the southward migration network of greater white‐fronted geese, the effective resistance increased faster when sites were removed in order of decreasing node resistance or degree (Fig. 2d & Supporting Information). For most networks, site removal in order of degree of habitat loss yielded an effective resistance increase similar to random site removal. However, in the southward migration network of swan geese, effective resistance increased more rapidly upon site removal in order of habitat loss compared with all other removal orders (Fig. 2b). The northward migration network of swan geese showed the opposite pattern; random site removal yielded a more rapid increase in effective resistance.

### Site Importance

**Figure 3:**
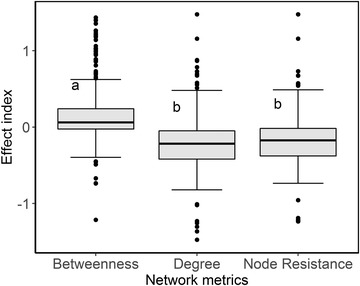
Differences in the effect indices of betweenness, degree, and node resistance in the migration networks of tracked birds (letters, identical groups as determined by the multiple comparison test at p = 0.05; boxes, first and third quartiles; line within boxes, median; whiskers, minimum and maximum; circles, outliers).

**Figure 4:**
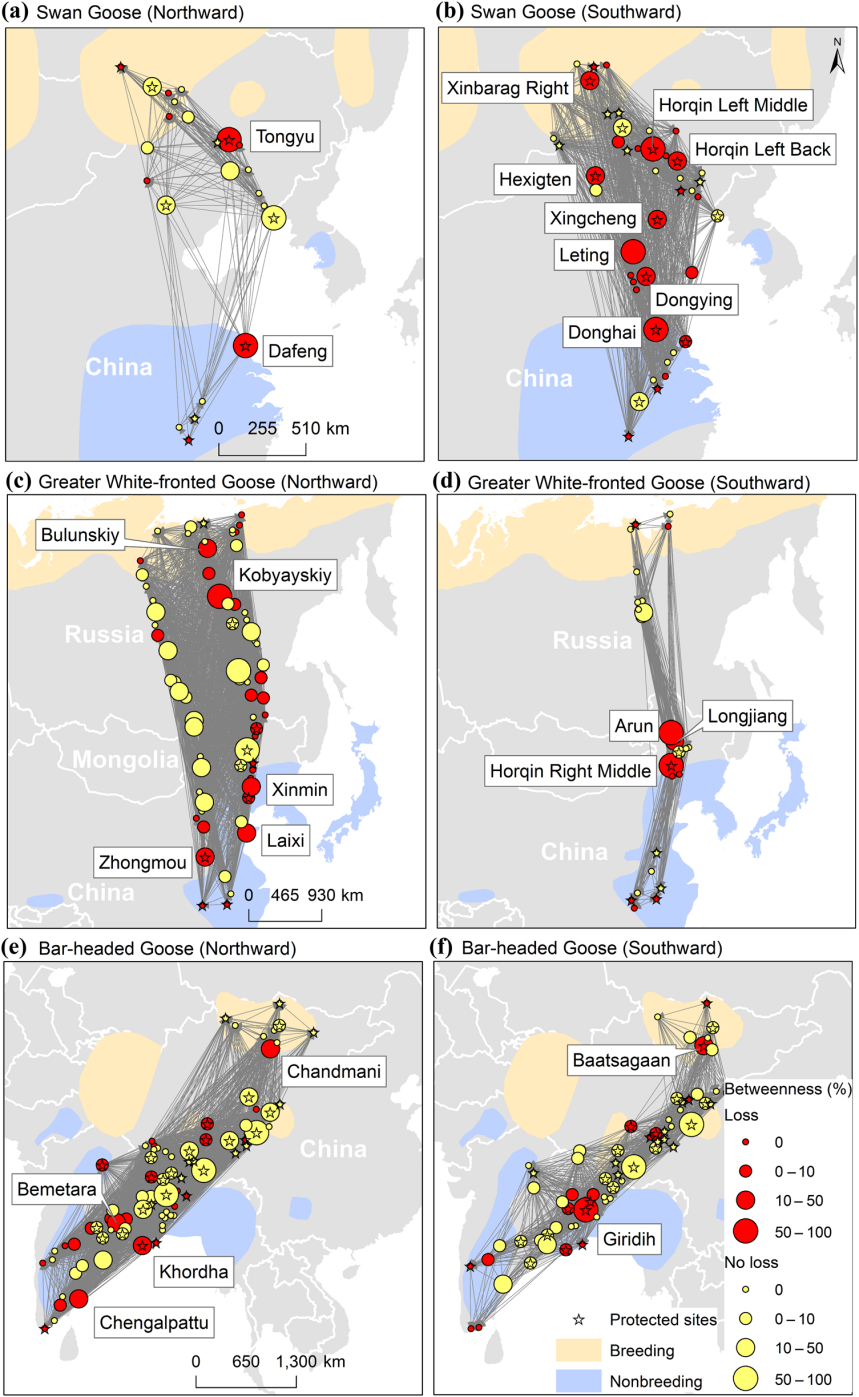
Migration networks and locations of crucial stopover sites for 4 bird species. The betweenness of each stopover was normalized via division by the highest betweenness value in the corresponding network (circle size, normalized betweenness; red, sites with habitat loss from 1992 to 2015; yellow, sites without habitat loss; stars, protected sites). Crucial stopover sites have a normalized betweenness of ≥10% and habitat loss (mean = 7.8%; 95% CI 4.1%) and are identified by counties in China, districts in Russia, sums in Mongolia, and taluks in India. Species’ breeding and nonbreeding ranges are from Bird Distribution Maps of the World (version 5.0) produced by Birdlife International (birdlife.org).

Effect indices significantly differed between different network metrics (Kruskal–Wallis test, *χ* ^2^ = 98.0, df = 2, *p* < 0.001). Betweenness had a significantly higher effect index than degree and node resistance (Fig. 3). Therefore, we defined a site's importance for maintaining network connectivity based on betweenness. Sites were defined as crucial if they showed high relative betweenness (≥10%) and were also likely to be removed from the network (i.e., subject to habitat loss) (mean = 7.8%, 95% CI 4.1%) (Fig. 4).

We identified 24 crucial sites: China 16, India 4, Mongolia 2, and Russia 2. The sites in the Northeast China Plain (i.e., Tongyu, Xingcheng, Horqin left back banner, Hexigten, Horqin left middle banner, Xinmin, Horqin right middle banner, Longjiang, and Linxi) played important roles in the migration networks of swan geese and greater white‐fronted geese. Of the 24 crucial sites, 8 were in coastal regions of China (Dafeng, Donghai, Leting, Dongying, Xingcheng, and Laixi) or India (Chengalpattu and Khordha). Among sites with high betweenness in the southward migration network of swan geese, 80% showed habitat loss (1.5–40.0%), and 50% of the crucial sites were in the coastal region of China. Among all stopover sites, 66% were not protected, and 10 crucial sites were not designated as protected areas (Fig. 1b & Supporting Information).

## Discussion

A well‐connected migration network of well‐protected sites can decrease migration costs and risks and thus facilitate bird migratory movements and increase migration success (Merken et al. 2015). Species that occupy large and robust migration networks have more alternative efficient routes and are thus better able to cope with natural and human‐driven environmental changes (Xu et al. 2019b). The migration network structure of waterfowl in the East Asian–Australasian Flyway partly explains the population size fluctuations of these migratory birds because population sizes decrease with losses of migration network functional connectivity (Xu et al. 2019a). We quantitatively evaluated the robustness of migratory birds’ migration network and identified crucial stopover sites in terms of contribution to network connectivity and degree of habitat loss. Regional policy makers could apply our analytical framework when establishing conservation priorities to decrease the risk of migration network collapse and to monitor policy implementation by local authorities.

Many studies have examined the importance of nodes within a network, and multiple indices have been proposed for quantifying the contributions of individual nodes toward network connectivity (Freeman 1979; Newman 2005; Opsahl et al. 2010). However, when identifying crucial sites, the utilized index should reflect their contributions to migration‐network connectivity (e.g., betweenness) as well as account for the ecological processes and mechanisms. Theoretical works have constructed simplified full‐annual‐cycle models for bird migration to investigate bird population dynamics under habitat loss (Weber et al. 1999; Iwamura et al. 2013; Hostetler et al. 2015). However, these theoretical models do not consider the complexities of the spatial configurations of species‐specific migration networks, such as differences in flyway broadness, migration distances, sizes of breeding ranges relative to nonbreeding ones, and alternative migration routes (Morrison et al. 2013; Gilroy et al. 2016; Xu et al. 2019b). Our model accounted for these features based on the empirical configuration of species‐specific migration networks and defined crucial stopover sites in existing networks, which is crucially important for enabling successful migration. Further research could be performed using our framework, modifying the algorithms and assumptions that define the probability of between‐site movements. We only accounted for upper limits of nonstop flights, energetic terms, and migratory directions, but other factors may also influence the cost of long‐distance flights (Dokter et al. 2018), such as increased predation risk or disturbance and the costs of searching and settling when making multiple stopovers. When sample sizes permit, it is possible to empirically estimate the probability of traveling different distances based on tracking data (Dhanjal‐Adams et al. 2017).

Comparing the effects of node removal in the order of different metrics revealed betweenness as the most important factor identifying important stepping‐stones. Betweenness measures the importance of a site in facilitating movement throughout the network (Newman 2005), and removing nodes with high betweenness rapidly disconnects a network (van Mieghem et al. 2017). Sites with high betweenness are necessary steps in multiple least‐cost paths, such that their removal may force migrants to take suboptimal paths. Removal or degradation of sites with high betweenness can impede successful migration. The importance of crucial sites in movement networks has also been reported for some forest bird species, whose mobility for range shift and long‐distance dispersal can be sharply reduced by the loss of one critical stepping‐stone site (Saura et al. 2014). Future studies could analyze the availability and quality of the currently unused suboptimal alternative paths, which may serve as new migration routes that could prevent network collapse.

Other network metrics (i.e., node resistance and degree) may also play important roles in identifying pivotal stepping‐stones in migration networks for some species. We found that node resistance and degree outperformed betweenness in the southward migration network of greater white‐fronted geese. However, in some smaller networks (e.g., the northward migration network of whooper swans), node removal in the orders of node resistance and degree reduced network connectivity more slowly than random removal. Therefore, node resistance and degree should be used in comparison with betweenness, which is a more general metric that can be applied to designating prioritized conservation efforts for migration networks of various species.

The removal of sites with a high degree of habitat loss did not increase the effective resistance more than random site removal, suggesting that migration networks were resistant to patterns of habitat loss (from 1992 to 2015). Habitat loss had occurred in only some of the critical sites with a high contribution to network connectivity. However, removal of only a few sites with high betweenness can rapidly decrease network connectivity and trigger sudden collapse. These results are in agreement with theoretical simulations showing that high levels of habitat loss at random sites have less of an impact on migratory species than low levels of habitat loss at critical sites (Runge et al. 2014). Loss of specific sites in a migration network (e.g., Bohai Bay in eastern China [Shimazaki et al. 2004] and the Yellow Sea tidal mudflat [Studds et al. 2017]) may isolate breeding from nonbreeding sites, and trigger rapid population declines in migratory birds (Xu et al. 2019a). Therefore, the selection of crucial conservation areas for migratory species must account for both the severity of habitat degradation and the site's context within the species’ network.

Migration‐network connectivity rapidly decreased when a network comprised a small number of sites. Networks are stable when migratory birds have plentiful alternative sites, but become more vulnerable following successive loss of sites. Unfortunately, some forms of habitat change cannot be detected by land‐cover classification (e.g., water pollution and poaching). However, the presently detected pattern of habitat loss was in agreement with previous studies showing that coastal regions and inland natural wetlands in eastern China have severe habitat loss due to rapid urbanization and sea‐level rise (Niu et al. 2012; Xu et al. 2019b). We also demonstrated that other sites in the migration networks were gaining habitat area, for example, in western China, because artificial wetlands were created in the form of fish farms and reservoirs (Niu et al. 2012). These increased alternatives can improve migration movement flexibility, boost network resilience, and subsequently mitigate population declines of migratory birds subjected to environmental change (Gilroy et al. 2016; Patchett et al. 2018). Overall, preventing habitat degradation and adding artificial habitats are essential for preventing migration network collapse, especially in locations with high betweenness.

Because we investigated identical network structures for different species, our results quantified the conservation needs of certain species and corresponding sites. Apart from the migration networks of whooper swans, which might be biased by the small sample size (Supporting Information), the most vulnerable migration network was that of swan geese. The swan goose is categorized as a vulnerable species with relatively small population sizes and limited distribution (Birdlife International 2016), and this species has already lost habitat area in important stepping stones in its networks over the past 2 decades. Its network integrity is impaired to the extent that it is now close to collapsing and holds fewer alternative routes compared with a random network. As shown in Fig. 2, compared with their southward migration network, the northward migration network of swan geese is even more vulnerable to collapse because the loss of only 14 sites will lead to network collapse. The Northeast China Plain and coastal regions in China contain critical sites (Studds et al. 2017; Si et al. 2018), many of which are currently unprotected (Fig. 1b). This highlights the need for urgent conservation efforts at the province level because protection at lower administrative levels reportedly has little or no effect (Zhang et al. 2015). Although the population numbers of greater white‐fronted geese are declining in the East Asian–Australasian Flyway (Zhang et al. 2015), the numbers of bar‐headed geese in the Central Asian Flyway may actually be increasing. Our network integrity analysis appears to indicate that their migratory networks are sufficiently robust (Fig. 2). However, concerns are raised by the rapid breakdown of the southward migration network of greater white‐fronted geese upon node removal in the order of decreasing node resistance.

Our results provide compelling evidence that destroying stopover sites with high betweenness values rapidly reduced migration‐network connectivity. Node resistance and degree were also important metrics for specific networks (e.g., the southward migration network for greater white‐fronted geese). Our analysis was based on tracked individuals, which constitute only a small fraction of the total species population. Additional data collection may lead to identification of other crucial sites for these species across their range.

Our results provide insights for evaluating migration network robustness, which is useful for guiding the rational allocation of conservation efforts and funds. To effectively conserve migratory species, we suggest that policy makers emphasize the designation and management of crucial sites that strongly contribute to the migration network's connectivity and exhibit a high degree of habitat loss. Among the crucial sites identified in this study, 42% are not currently protected (Supporting Information). These sites could be prioritized for listing under the flyway site networks, for example, of the EAAFP.

Our analytical framework involves a network approach and can be applied to help predict and prevent migration network collapse and thus to provide guidance for regional policy makers. Our approach could be applied to other criteria in addition to those we used to identify important habitats that need protection. For example, the current Ramsar Sites Criteria define a wetland as internationally important if it exhibits high biodiversity, if vulnerable species are observed, or if it is a special wetland type (Wetland International 2012). However, these criteria do not account for the wetland's importance in terms of the migration network's connectivity.

## Supporting information

A summary of the tracking data we used (Appendix S1), capture sites (Appendix S2), migration lags of study species (Appendices S4 & S5), overall network metrics of migration networks (Appendices S6–S8), information regarding the identified crucial sites (Appendix S9), plots for the sensitivity analysis of the effect of number of tracks on site detection (Appendix S3), and species‐specific comparison among network metrics (Appendix S10) are available online. The authors are solely responsible for the content and functionality of these materials. Queries (other than regarding the absence of the material) should be directed to the corresponding author.
