# Supplementary material for: A network approach to prioritize conservation efforts for migratory birds
Source: Conserv Biol. 2019 Aug 16;34(2):416–26. doi: 10.1111/cobi.13383 (PMC7154769; doi:10.1111/cobi.13383)
Supplement: Supplementary file 1 — A summary of the tracking data we used (Appendix S1), capture sites (Appendix S2), migration lags of study species (Appendices S4 & S5), overall network metrics of migration networks (Appendices S6–S8), information regarding the identified crucial sites (Appendix S9), plots for the sensitivity analysis of the effect of number of tracks on site detection (Appendix S3), and species‐specific comparison among network metrics (Appendix S10) are available online. The authors are solely responsible for the content and functionality of these materials. Queries (other than regarding the absence of the material) should be directed to the corresponding author. [file COBI-34-416-s001.docx]

**A network approach to prioritize conservation efforts for migratory birds**

**Supporting information**

**Appendix S1** A summary of tracking data, with species, scientific name, number of spring and autumn tracks and number of tracked birds. Sources of the tracking data were described with study name in movebank.org.

| Common name | Scientific name | Flyway | Northward tracks | Southward tracks | Total individuals | Data source |
| --- | --- | --- | --- | --- | --- | --- |
| Swan Goose | *A. cygnoides* | EAAF | 7 | 42 | 81 | 2015 Tsinghua Waterfowl  FAO-USGS_Mongolia-East FAO-USGS Mongolia-2014 |
| Greater White-fronted Goose | *A. albifrons* | EAAF | 17 | 14 | 54 | 2015 Tsinghua Waterfowl |
| Whooper Swan | *C. cygnus* | EAAF | 3 | 7 | 10 | FAO-USGS_Mongolia-East |
| Bar-headed Goose | *A. indicus* | CA | 36 | 45 | 93 | FAO-USGS_China-Qinghai  FAO-USGS_India-2009  USGS_India-Nepal  FAO-USGS_Mongolia-West |

**Appendix S2** Information of capture sites for the tracked individuals.

| Source study | Species | Country | Site | Latitude | Longitude | Seasonal | Data  Period |
| --- | --- | --- | --- | --- | --- | --- | --- |
| 2015 Tsinghua Waterfowl | *A. albifrons;*  *A. cygnoides* | China | Poyang Lake | 29.1° N | 116.3° E | Non-breeding | 2015-2018 |
| 2015 Tsinghua Waterfowl | *A. cygnoides* | China | Hulun Lake | 48.3° N | 117.4° E | Moulting | 2016-2018 |
| FAO-USGS  Mongolia-East | *A. cygnoides;*  *C. cygnus* | Mongolia | Mongol  Daguur | 49.7° N | 114.7° E | Moulting | 2006-2009 |
| FAO-USGS Mongolia-2014 | *A. cygnoides* | Mongolia | Mongol  Daguur | 49.6° N | 115.6° E | Moulting | 2014-2016 |
| FAO-USGS  China-Qinghai | *A. indicus* | China | Qinghai Lake | 37.1° N | 99.4° E | Breeding | 2007-2013 |
| FAO-USGS  India-2009 | *A. indicus* | India | Chilika Lake | 19.7° N | 85.3° E | Non-breeding | 2008-2010 |
| USGS  India-Nepal | *A. indicus* | Nepal | Chitwan National Park | 27.7° N | 84.3° E | Non-breeding | 2005 |
| FAO-USGS  Mongolia-West | *A. indicus* | Mongolia | Terkhiin Tsagaan Lake | 48.2° N | 99.7° E | Moulting | 2008-2010 |

**Appendix S3** Sensitivity analysis of the effect of the number of tracks on detection of sites for (a) southward migration of swan Geese; (b) southward migration of bar-headed geese.


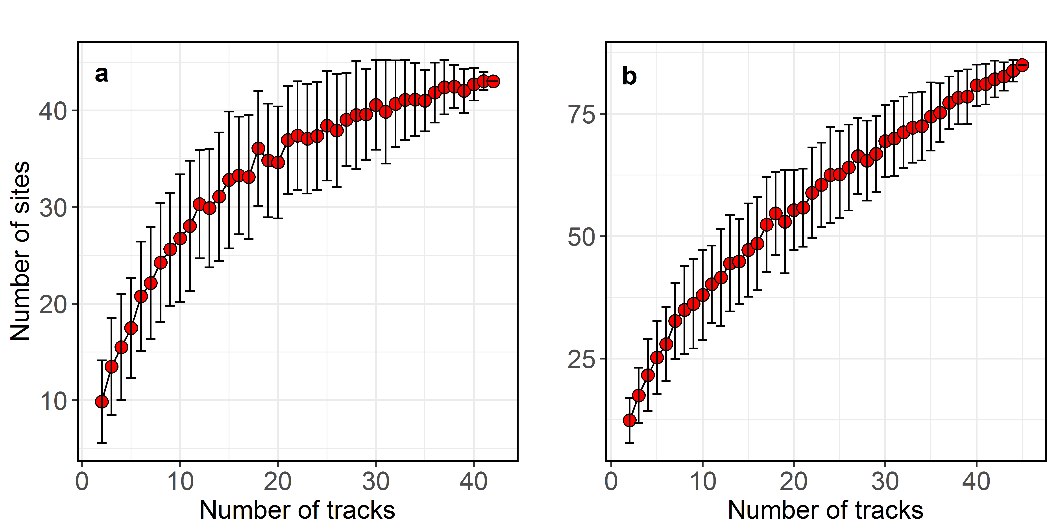


**Appendix S4** Median, maximum, and 95% confidence interval for median migration lag of autumn and spring migrations of Bar-headed Goose, Greater White-fronted Goose, Swan Goose, and Whooper Swan in kilometers.

| Species | Direction | Median | Maximum | 95% CI (km) | | Sample size |
| --- | --- | --- | --- | --- | --- | --- |
|  |  |  |  | Upper | Lower |  |
| Swan Goose | Northward | 168 | 1396 | 335 | 118 | 36 |
|  | Southward | 168 | 2063 | 227 | 125 | 215 |
| Greater White-fronted Goose | Northward | 217 | 3018 | 299 | 148 | 147 |
|  | Southward | 1430 | 3180 | 1790 | 350 | 40 |
| Whooper Swan | Northward | 261 | 1152 | 445 | 145 | 16 |
|  | Southward | 411 | 885 | 606 | 193 | 22 |
| Bar-headed Goose | Northward | 194 | 2295 | 229 | 139 | 189 |
|  | Southward | 202 | 1369 | 231 | 185 | 118 |

**Appendix S5** Violin plots of migration lags (box plots with rotated kernel density plots on each side) in northward and southward migration for bar-headed geese (BHG), greater white-fronted geese (GWFG), swan geese (SG), and whooper swans (WS). The ‘migration lag’ is the birds’ travel distance from one site to the next.


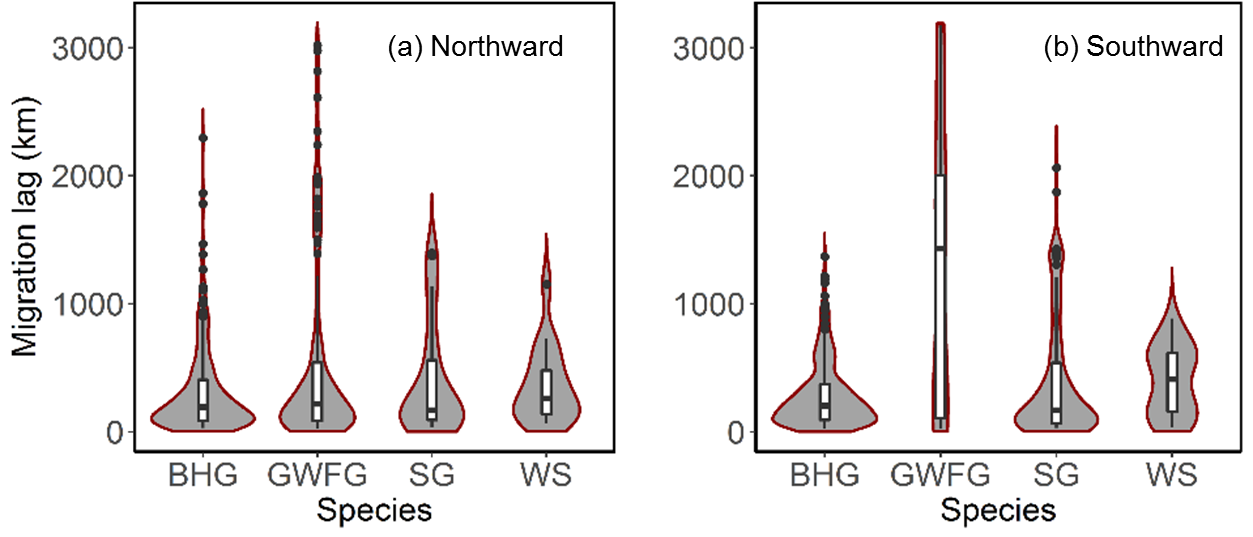


## **Appendix S6 Overall network metrics**

**Methods.** We calculated several overall networks to indicate the connectivity and characteristics of the studied migration networks. These overall network metrics include the number of nodes, network size, graph density, graph diameter, modularity, number of alternative routes and shortest path length between the southernmost non-breeding and northernmost breeding sites (hereafter as path length).

The ‘size of a network’ is the total number of connections between nodes which indicates the total number of sites used by the birds (Wasserman & Faust 1994). ‘size of a network’ indicates the total number of possible steps the bird can take in the migration networks. The ‘graph density’ is the proportion of actual connections in all possible connections in a network (Wasserman & Faust 1994). The higher the ‘graph density’, the more intensively the sites are connected in the migration networks. The ‘graph diameter’ is minimum number of steps for the bird to travel between the two farthest sites (breeding and non-breeding sites) in the network. We measured weighted path lengths between each pair of nodes in these networks by the Dijkstra algorithm (Dijkstra 1959). ‘Path length’ is calculated by the weighted path length between the two farthest sites in the network which measures the lowest costs the bird has to make to move between the southernmost non-breeding and northernmost breeding sites. . ‘Modularity’ measures the degree to which a network were divided into smaller modules. Modules were formed by a group of nodes in the network, maximizing the connectivity within each of the modules and minimizing the connectivity between different modules (Newman 2006). The higher the modularity, the denser the connections between the nodes within modules, but the sparser the ties connecting these different modules (Newman 2006). We measured the number of alternative routes between the southernmost non-breeding and northernmost breeding sites by analysing the adjacency matrix of sites in a network (O'sullivan & Unwin 2014; Shimazaki et al. 2004).

We generated 999 random networks whose nodes were randomly generated within the extents of the species’ movement as calculated from the available tracking data. The random networks had the same number of nodes, and the weighted connections between these nodes were calculated with the same methods as for the observed migration networks. We compared graph density, modularity, number of alternative routes and path length of the migration networks with 999 random networks which were used as null models (Appendix S8) to indicate how the empirical migration networks are different from the random configurations.

**Results.** The diameters of most migration networks of the study species was 2 (Appendix S7), which means at least one stopover site is required for a successful travel between their northernmost breeding sites and southernmost non-breeding sites. However, for bar-headed geese in southward migration, at least three stopover sites are required for completing the full migration. bar-headed geese had northward and southward migration networks with a higher graph density and number of alternative routes, a lower path length and modularity than the random networks (Appendix S8). The southward migration network of greater white-fronted geese had a lower graph density and higher modularity compared to the random ones. Although their northward migration network had a higher graph density compared to the random networks, its path length was higher than the random ones. The southward migration network of swan geese had a higher graph density and a lower path length, but a lower number of alternative routes than the random networks.

**References**

Dijkstra EW. 1959. A note on two problems in connexion with graphs. Numerische Mathematik 1:269-271.

Newman MEJ. 2006. Modularity and community structure in networks. Proceedings of the National Academy of Sciences of the United States of America 103:8577-8582.

O'sullivan D and Unwin D. 2014. Geographic information analysis. John Wiley & Sons.

Shimazaki H, Tamura M, Darman Y, Andronov V, Parilov MP, Nagendran M, Higuchi H. 2004. Network analysis of potential migration routes for Oriental White Storks (*Ciconia boyciana*). Ecological Research 19:683-698.

Wasserman S and Faust K. 1994. Social network analysis: Methods and applications. Cambridge university press.

**Appendix S7** Overall network metrics of migration networks of each species per season.

| Species | Swan Goose | |  | Greater White-fronted Goose | |  | Whooper Swan | |  | Bar-headed Goose | |
| --- | --- | --- | --- | --- | --- | --- | --- | --- | --- | --- | --- |
| Network metric | North-ward | South-ward |  | North-ward | South-ward |  | North-ward | South-ward |  | North-ward | South-ward |
| Number of nodes | 23 | 45 |  | 72 | 27 |  | 15 | 13 |  | 81 | 67 |
| Network size | 190 | 987 |  | 2226 | 272 |  | 50 | 48 |  | 2574 | 1177 |
| Graph density | 0.4 | 0.5 |  | 0.4 | 0.4 |  | 0.2 | 0.3 |  | 0.4 | 0.3 |
| Graph diameter | 2 | 2 |  | 2 | 2 |  | 4 | 4 |  | 2 | 4 |
| Modularity | 0.4 | 0.4 |  | 0.5 | 0.2 |  | 0.4 | 0.1 |  | 0.4 | 0.4 |
| Path length | 1.1E-02 | 8.9E-04 |  | 7.5E-04 | 5.9E-01 |  | 8.6E-01 | 1.7E+00 |  | 8.0E-04 | 4.3E-02 |
| Routes | 2.0E+06 | 8.8E+12 |  | 1.2E+21 | 3.4E+07 |  | 1.6E+03 | 4.9E+02 |  | 6.0E+23 | 3.7E+19 |
| Tracks | 7 | 42 |  | 17 | 14 |  | 3 | 7 |  | 36 | 45 |

**Appendix S8** Comparison of empirical migration networks with random networks. Red triangles are the value of the corresponding metrics in the empirical migration networks, and the grey circles and bars represent mean and standard deviation of these corresponding metrics of the 999 random networks. Species names are abbreviated as BHG (bar-headed goose), GWFG (greater white-fronted goose), SG (swan goose), and WS (whooper swan), followed by a letter “A” or “S” which refers to southward and northward migration, respectively.

**
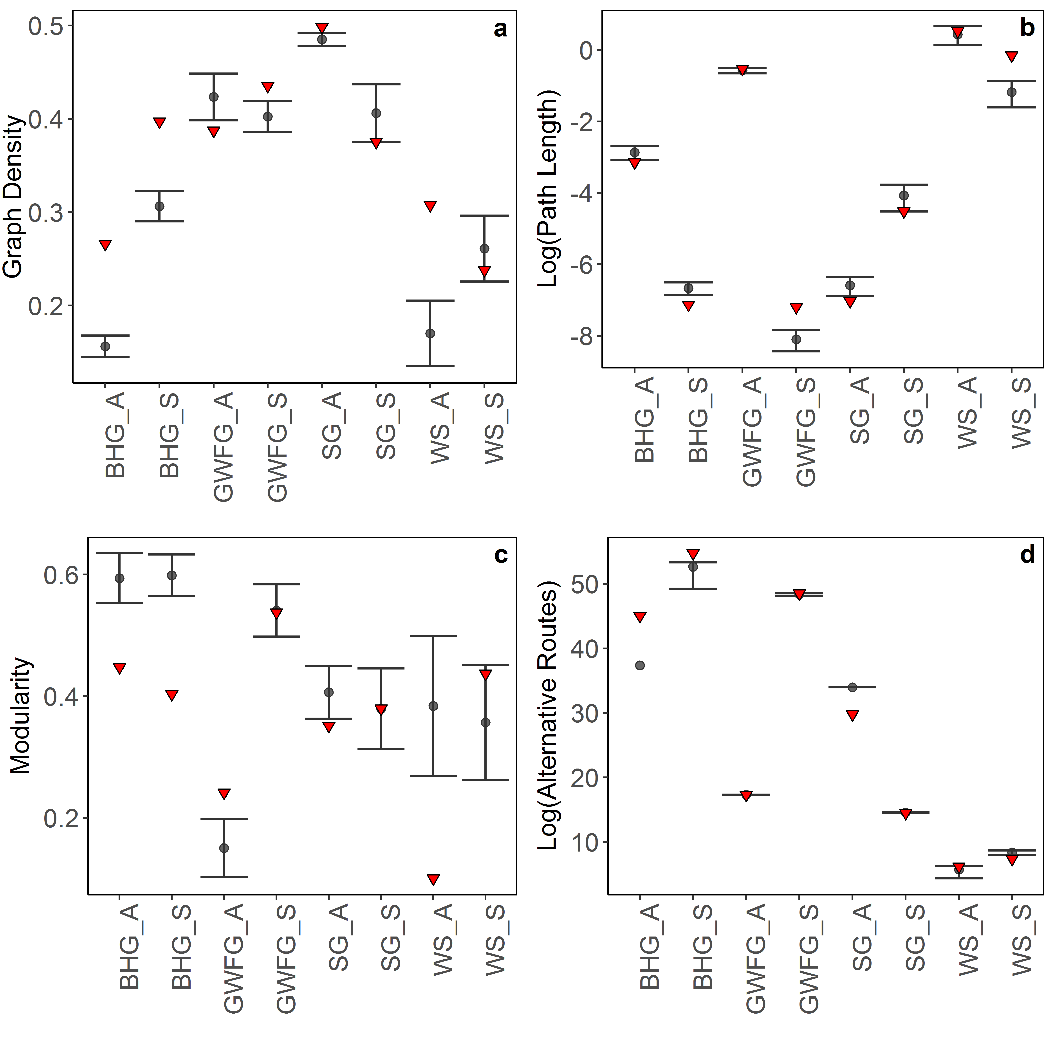
**

**Appendix S9** Differences in the effect indices of betweenness, degree, and node resistance for each studied migration network. The letters on the boxes represent the identical groups as identified by the multiple comparison test at p=0.05. a, c, e are northward migration networks of swan geese, greater white-fronted geese, and bar-headed geese, respectively; b, d, f are southward migration networks of swan geese, greater white-fronted geese, and bar-headed geese, respectively.


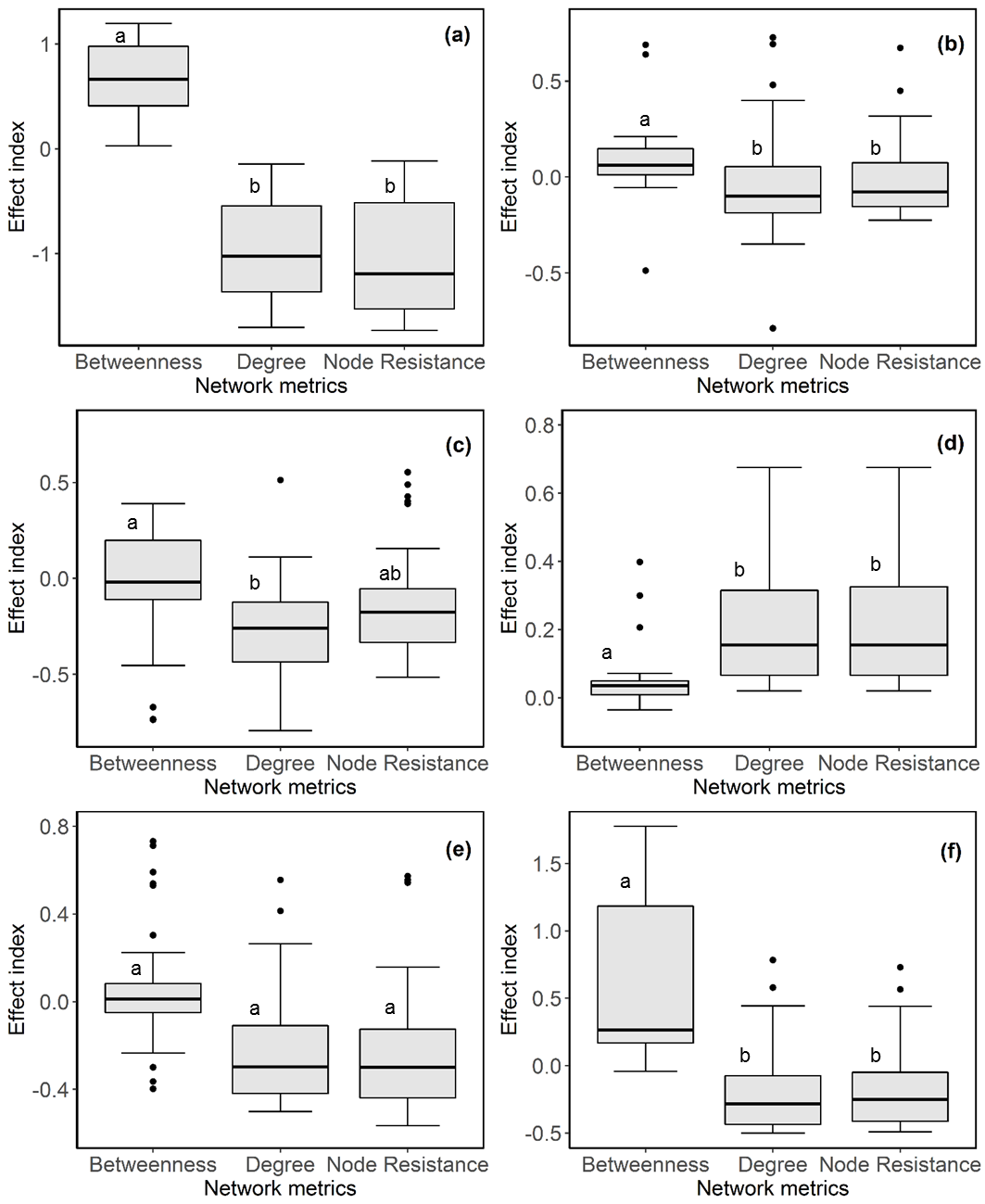


**Appendix S10** Information of identified crucial sites. Species names are abbreviated as BHG (bar-headed goose), GWFG (greater white-fronted goose), SG (swan goose), and WS (whooper swan).

| ID | Name | Country | Latitude | Longitude | Habitat loss | Protection | Species | Direction |
| --- | --- | --- | --- | --- | --- | --- | --- | --- |
| 1 | Dafeng | China | 33.5093 | 120.6183 | 1.1% | Yes | SG | Northward |
| 2 | Tongyu | China | 44.6616 | 122.5036 | 16.1% | Yes | SG | Northward |
| 3 | Donghai | China | 34.4257 | 119.0113 | 40.0% | Yes | SG | Southward |
| 4 | Dongying | China | 37.3762 | 118.9935 | 5.5% | Yes | SG | Southward |
| 5 | Leting | China | 38.8283 | 118.4182 | 6.3% | No | SG | Southward |
| 6 | Xingcheng | China | 40.3308 | 120.5196 | 14.8% | Yes | SG | Southward |
| 7 | Horqin Left Back | China | 43.2624 | 122.8737 | 2.5% | Yes | SG | Southward |
| 8 | Hexigten | China | 43.2912 | 116.5685 | 2.8% | Yes | SG | Southward |
| 9 | Horqin Left Middle | China | 44.1948 | 121.2612 | 9.5% | Yes | SG | Southward |
| 10 | Xinbarag Right | China | 48.5478 | 117.2547 | 1.5% | Yes | SG | Southward |
| 11 | Zhongmou | China | 34.9192 | 113.9925 | 19.4% | Yes | GWFG | Northward |
| 12 | Laixi | China | 36.9554 | 120.4506 | 10.0% | No | GWFG | Northward |
| 13 | Xinmin | China | 42.2175 | 122.6565 | 0.3% | No | GWFG | Northward |
| 14 | Kobyayskiy | Russia | 64.8114 | 125.6569 | 9.1% | No | GWFG | Northward |
| 15 | Bulunskiy | Russia | 70.2565 | 125.9493 | 0.2% | No | GWFG | Northward |
| 16 | Horqin Right Middle | China | 44.5601 | 121.9112 | 7.4% | No | GWFG | Southward |
| 17 | Longjiang | China | 47.3683 | 123.4235 | 0.6% | No | GWFG | Southward |
| 18 | Arun | China | 48.4606 | 123.1705 | 0.2% | No | GWFG | Southward |
| 19 | Chengalpattu | India | 12.4630 | 80.1382 | 47.7% | No | BHG | Northward |
| 20 | Khordha | India | 19.8819 | 85.2713 | 6.8% | Yes | BHG | Northward |
| 21 | Bemetara | India | 21.5390 | 81.5831 | 3.0% | No | BHG | Northward |
| 22 | Chandmani | Mongolia | 45.2256 | 98.2113 | 4.9% | No | BHG | Northward |
| 23 | Giridih | India | 24.4330 | 86.2508 | 3.6% | Yes | BHG | Southward |
| 24 | Baatsagaan | Mongolia | 45.7136 | 99.3265 | 4.3% | Yes | BHG | Southward |
